# MEASURING COGNITION IN NSHAP USING MULTIMODE DATA COLLECTION

**DOI:** 10.1093/geroni/igad104.0236

**Published:** 2023-12-21

**Authors:** Kelly Pudelek, Henrique Ochoa Scussiatto, L Philip Schumm, Kristen Wroblewski, Selena Zhong, Meiyi Li

**Affiliations:** NORC at the University of Chicago, Chicago, Illinois, United States; University of Chicago, Chicago, Illinois, United States; University of Chicago, Chicago, Illinois, United States; University of Chicago, Chicago, Illinois, United States; NORC at the University of Chicago, Chicago, Illinois, United States; University of Wisconsin-Madison, Madison, Wisconsin, United States

## Abstract

The National Social Life, Health, and Aging Project (NSHAP) is a U.S. nationally representative, omnibus study of social relationships and health among home-dwelling older adults. Cognitive function is a key focus, both as an outcome and also because it is implicated in mechanisms by which social relationships affect and are affected by health trajectories. Data collection started in 2005, and respondents have been re-interviewed in 2010, 2015, and 2022–3. Starting in 2010, NSHAP administers a survey-adapted version of the Montreal Cognitive Assessment (MoCA-SA). While Rounds 1–3 were conducted in the home, Round 4 utilized three remote modes (web, phone and PAPI, in that order) in addition to in-home interviews. Respondents were assigned to remote data collection initially if they were judged to be likely to respond based on prior information, otherwise they were assigned to in-home; a random subset of 400 initially assigned to remote were shifted to the in-home group to facilitate examination of possible mode effects on response. Each mode necessarily utilized a slightly different subset of the MoCA-SA items based on feasibility; these were augmented by items from the Rush Alzheimer’s Disease Center’s (RADC) cognition battery that could be administered via the web or PAPI. This presentation will describe our analytic strategy for harmonizing across modes and generating an overall measure of cognitive function, as well as preliminary results for Round 4. Our approach utilizes a bi-factor item response model calibrated to each mode, and is applicable to data from other studies.

